# Effects of neuromodulation on cognitive performance in individuals exhibiting addictive behaviour: a systematic review protocol

**DOI:** 10.1186/s13643-018-0749-y

**Published:** 2018-06-26

**Authors:** Katherine R. Naish, Lana Vedelago, James MacKillop, Michael Amlung

**Affiliations:** 1 Peter Boris Centre for Addictions Research, Department of Psychiatry and Behavioural Neurosciences, St. Joseph’s Healthcare Hamilton and McMaster University, 100 West 5th Street, Hamilton, Ontario L8P 3R2 Canada; 2 Centre for Medicinal Cannabis Research, Michael G. DeGroote School of Medicine, McMaster University, L8S 4K1 Hamilton, Ontario Canada; 3 Department for Psychiatry and Behavioural Neurosciences, St. Joseph’s Healthcare Hamilton, 100 West 5th, L8N 3K7 Hamilton, Ontario Canada

**Keywords:** Transcranial direct current stimulation, Transcranial magnetic stimulation, Brain stimulation, Neuromodulation, Dorsolateral prefrontal cortex, Addiction, Substance use disorder, Impulsivity, Risk-taking, Delay discounting

## Abstract

**Background:**

Individuals with substance use and addictive disorders often display greater risk-taking behaviour, higher impulsivity, and altered reward processing compared to individuals without these disorders. While it is not known whether cognitive biases precede or result from addictive behaviour, they likely influence addiction-related decision-making, and may facilitate pathological behaviour. There is evidence that cognitive functions—including those shown to be altered in substance use and addictive disorders—can be influenced by neuromodulation techniques (specifically, transcranial direct current stimulation and transcranial magnetic stimulation). Much of this work has been conducted in healthy populations, however, making it unclear whether these methods can be used effectively to modulate cognitive functioning in individuals with substance use and addictive disorders. The purpose of the current review is to shed light on the potential effectiveness and feasibility of neuromodulation as a means to improve cognitive deficits in substance use disorders.

**Methods:**

The review will identify and evaluate studies that have examined the effects of transcranial direct current stimulation (tDCS) or transcranial magnetic stimulation (TMS) on cognitive task performance in individuals with chronic substance use or dependence. Relevant studies will be identified through searches in PubMed, PsycINFO, Scopus, and Embase, and narrative review will be used to explore evidence that these techniques can be used successfully to modulate cognitive performance in populations exhibiting addictive behaviour. Assessing individual cognitive domains in turn (e.g. risk-taking, impulsivity, attention), we will critically evaluate the validity and reliability of relevant studies and draw conclusions about the strength of evidence for effective use of neuromodulation in that domain. This protocol is not yet registered with PROSPERO.

**Discussion:**

To determine whether neuromodulation holds promise as an effective treatment for neurocognitive deficits in substance use and addictive disorders, it is essential to look carefully at previous studies using this approach in addiction samples. This review will provide an objective and informative description of what is currently known about the efficacy of these techniques, shed light on the feasibility and potential challenges of using neuromodulation in individuals who exhibit addictive behaviour, and identify the most valuable next steps for future research.

## Background

Substance use disorders are among the 20 leading causes of disability and account for around 0.4% of all deaths globally [31]. In addition, gambling disorder is associated with reduced quality of life [18], cognitive deficits [29], and high levels of suicidality [22]. The growing impact of substance use and addictive disorders (referred to hereafter as ‘SUAD’) is particularly pronounced within high socio-demographic areas, where the number of years of life lost to substance abuse has increased by nearly 20% in the last two decades [28]. In addition to their direct impact on individuals’ psychological and physical health, SUAD often leads to a loss of employment and breakdown of social relationships, as obtaining and using the drug—or engaging in gambling—takes precedence over work, family, and other obligations. Given their increasing prevalence and considerable societal impact [27], establishing effective treatment approaches is of paramount importance.

Impaired control and risky behaviour related to use of a substance are hallmark features of SUAD [1]. Compared to individuals without SUAD, individuals who exhibit addictive behaviour also tend to show greater impulsivity [25], impaired decision-making [9], and heightened attention towards stimuli associated with their disorder [12]. In the former cases, increased impulsivity commonly manifests as greater preferences for smaller-immediate rewards over larger-later rewards, also known as delayed reward discounting [2, 23]. One candidate neural mechanism behind the cognitive deficits present in SUAD is dysfunction in the prefrontal cortex (PFC). Individuals with SUAD commonly show differences from controls in PFC responses to both substance- and non-substance-related reward cues, with responses in this region sometimes predicting craving and SUAD severity (for a review, see [14]). Because the PFC is also implicated in many of the cognitive functions that are impaired in SUAD [8, 13], disruption in this area might underlie the cognitive impairments observed in these disorders. Although it is not clear whether these characteristics develop as a result of, or precede, addictive behaviour [16], modification of such cognitive biases can in fact reduce substance use behaviours [3, 4, 10, 30].

In healthy individuals, performance on cognitive tasks can be influenced by modulating cortical activity using non-invasive neuromodulation techniques. Transcranial direct current stimulation (tDCS) and transcranial magnetic stimulation (TMS) are methods of modulating brain activity by applying or inducing an electrical current over the scalp. TDCS works by applying a continuous weak electrical current directly to the scalp, while TMS is used to deliver discrete magnetic pulses over the scalp, which induce electrical activity in underlying cortical tissue by means of electromagnetic induction. Studies have demonstrated changes in impulsivity and risky decision-making following application of tDCS or TMS over prefrontal regions in healthy controls (e.g. [5, 6, 11, 21]). Given their effects in healthy populations, these techniques have gained interest as potential methods of modulating cognitive processes in clinical populations, including individuals with SUAD.

In the past decade, researchers have examined the effects of neuromodulation on a range of outcome measures related to substance use. While some studies suggest that self-reported craving and consumption can be reduced using neuromodulation (for reviews, see [15, 19, 20]), the effects on *cognitive* measures are less clear. It is also important to note that the effects on craving and consumption are not uniform across every individual with a SUAD, so identifying potential cognitive mechanisms that underlie or predict responsivity could be a crucial step in developing neuromodulation as a therapy for addictive disorders. Thus, the aim of the current review is to examine the findings of studies that have investigated the use of tDCS or TMS in modifying cognitive performance in addictive populations. While we do not have sufficient grounds to make directional predictions about how neuromodulation affects cognitive functioning in individuals with SUAD, we anticipate that such individuals are likely to respond differently compared to individuals without SUAD, and that there will be considerable variability *within* SUAD populations. Because neuromodulation has its effects through changing activity in the brain, underlying differences in brain structure or function could affect cognitive changes associated with neuromodulation. Differences in the reward network and prefrontal regions in substance users compared to non-users [14]—in addition to baseline differences in cognitive performance—may well change how neuromodulation affects cognitive functioning in these individuals.

## Methods/design

### Search strategy

Relevant original research articles will be identified using PubMed, PsycINFO, Scopus, and Embase. These databases will be searched using the search terms shown in Table 1. To identify relevant articles that were not captured by our initial search strategy, we will also conduct a manual search of the citations of included studies and other works of authors of included studies.

**Table 1:** Search terms used to identify relevant articles in electronic databases

1.	alcohol	16.	opiate
2.	alcoholic	17.	opioid
3.	cigarette	18.	heroin
4.	smoking	19.	benzodiazepine
5.	tobacco	20.	gambling
6.	crack	21.	drug
7.	cocaine	22.	addiction
8.	amphetamine	23.	substance
9.	methamphetamine	24.	‘behavioral addiction’
10.	stimulant	25.	‘substance use disorder’
11.	hallucinogen	26.	‘binge eating’
12.	THC	27.	‘food addiction’
13.	marijuana	28.	‘transcranial direct current stimulation’
14.	cannabis	29.	‘transcranial magnetic stimulation’
15.	sedative		
30.	Population search terms: 1 OR 2 OR 3 OR 4 OR 5 OR 6 OR 7 OR 8 OR 9 OR 10 OR 11 OR 12 OR 13 OR 14 OR 15 OR 16 OR 17 OR 18 OR 19 OR 20 OR 21 OR 22 OR 23 OR 24 OR 25 OR 26 OR 27		
31.	Neuromodulation search terms: 28 OR 29		
32.	Final search terms: 30 AND 31		

### Inclusion/exclusion of articles

To be included in the review, articles must meet the following criteria: (1) The article is an original research article published in a peer-reviewed journal (i.e. secondary reports—such as commentaries or review articles—will not be included); (2) the article is available in English; (3) the reported research investigates human participants; (4) the article reports the use of tDCS or TMS; (5) at least a subset of participants in the reported research are indicated as having a current or previous SUAD, sub-clinical substance use, or some level of dependence on a substance or behaviour; and (6) the reported research includes one or more measures of cognitive performance as outcome measures. Regarding our fifth criterion, our review will *not* be restricted to studies of individuals with a diagnosed SUAD; samples characterized by sub-clinical substance use or addictive behaviour (e.g. light alcohol or tobacco use, recreational gambling) will be included. In addition, we will not exclude studies of individuals with addictive behaviours secondary to, or concurrent with, another diagnosis or condition (e.g. schizophrenia; Parkinson’s disease). Although this could result in considerable heterogeneity between samples, including a range of addictive behaviours will allow us to capture any differences between individuals with different levels of dependence and use severity. Depending on the number of studies we find that report on individuals with diagnosed SUAD, sub-clinical substance use or dependence, and those with or without comorbid conditions, we will conduct secondary analyses to address differences between effects of neuromodulation in these groups. Regarding our sixth criterion, measures of cognitive performance can be any measure of psychological processing, where performance is not reliant exclusively on sensory input processes (e.g. visual detection) or output processes (e.g. motor performance in the form of reaction times). For example, a simple reaction time task using a single arbitrary cue would not be considered a measure of cognitive performance, because responses are dependent solely on perception of the cue and speed of responding. However, a task assessing reaction times to two different types of stimuli (e.g. substance-related vs. non-substance-related) would be included, because differences in response times could result from differences in attention to the two stimulus types. The current review will include measures of processes such as memory, attention, language, and decision-making. Measures of craving or consumption (alone) will not warrant inclusion in the review.

### Article screening

Articles resulting from the initial searches will be uploaded to the online systematic review software, ‘Covidence’ [7]. The abstracts of all articles will then be reviewed by two of the authors (KRN and LV), each of whom will vote on whether the article should be excluded or retained for full-text review. When a paper’s eligibility for inclusion cannot be inferred from the abstract, the paper will be retained for full-text review. In cases where the two reviewers disagree on an article’s inclusion, a third reviewer (MA or JM) will be consulted. Articles that are retained following abstract screening will then be subject to full text review. Again, two reviewers will vote on each article’s inclusion, and the opinion of a third reviewer (MA or JM) will be sought in cases where the first two reviewers disagree. Cohen’s kappa coefficient will be calculated to assess the level of agreement between the two reviewers at both the abstract screening and full-text screening stages. In line with the Preferred Reporting Items for Systematic Reviews and Meta-Analyses (PRISMA) guidelines [24], the number of articles excluded at each stage, and reasons for exclusion, will be depicted in the final report through a flow diagram.

### Risk of bias and quality assessment

Each study included in the review will be assessed by two reviewers for potential biases that could threaten the validity or reliability of the reported data. Based on the Cochrane Collaboration’s tool for assessing risk of bias [17], included articles will be rated as ‘low’, ‘high’ or ‘unclear’ for performance bias, attrition bias, and reporting bias. Assessment of performance bias will include identifying whether the participants and the experimenter were blinded to the experimental condition. Attrition bias will be assessed by comparing the number of participants initially enrolled in a study to the number of participants whose data was included in the final analysis (i.e. determining the rate of participant exclusion or drop-out). To assess potential reporting bias, the reviewers will assess whether any pertinent statistics or analyses are unreported. Given the nature of the populations being studied, we do not believe that assessing risk of selection bias is appropriate for the proposed review. Our search criteria were designed to identify studies that examined individuals with clinical or sub-clinical substance use, so the data assessed will represent individuals of specific populations. The use of neuromodulation techniques will restrict the samples further, because safe administration of these methods requires participants to meet several inclusion criteria related to health and lifestyle. We therefore anticipate a selection bias in most of the studies that we review and will discuss this separately, rather than attempting to assess selection bias in a systematic way.

The quality of included studies will also be evaluated by assessing sample size and experimental design. In terms of experimental design, we will assess the following specific points: inclusion and quality of a control condition (e.g. sham stimulation), inclusion and quality of a control participant group, and inclusion and quality of baseline measures of cognitive performance.

### Review procedures

For all included studies, the following information will be recorded: publication details (authors, year of publication, journal), sample size, sample characteristics, inclusion and exclusion criteria for participants, type of neuromodulation (i.e. tDCS or TMS), neuromodulation parameters, type of cognitive measure, experimental design, data analyses performed, and the reported results. Table 2 lists specific information that will be recorded for sample characteristics, neuromodulation parameters, experimental design, and results. Any required information that is not reported in a paper (and cannot be calculated from other information in the paper) will be requested from the corresponding author via email. All information and data collected throughout the review will be organized and stored in Excel databases.

**Table 2:** Specific information to be recorded for every study included in the review. For studies that compare performance between two or more groups of participants, the sample characteristics will be recorded for each group separately. For continuous variables (e.g. age, level of substance use engagement), the mean, standard deviation, and range will be recorded. For discrete or categorical data (e.g. gender, diagnoses, treatment status), the number of participants in the sample who fall into each classification will be recorded

Information type	Details to be recorded
Sample characteristics	Age
	Gender
	SUAD diagnoses (if applicable); years of diagnosis
	Level of substance use or behaviour engagement/severity (e.g. number of cigarettes smoked per day; score on validated measure of addictive behaviour)
	Treatment status (number of participants in inpatient or outpatient treatment, seeking treatment, not seeking treatment)
	Substance use abstinence period before study; requirement to refrain from use before study
Neuromodulation parameters	Type of neuromodulation (i.e. tDCS, TMS); stimulation pattern/frequency for TMS (i.e. single-pulse, repetitive, theta-burst, patterned)
	Position of stimulating electrodes (tDCS) or coil (TMS)
	Stimulation intensity
	Stimulation duration
	Stimulation frequency (for tDCS and repetitive TMS)
	Number of sessions of stimulation; interval between sessions (if repeated sessions)
	Number of pulses; duration of stimulation trains and inter-train intervals (TMS)
	Electrode sizes (tDCS); coil type (TMS)
	Inclusion and details of sham condition
Experimental design	Within-subject or between-subject comparisons
	Single- or double-blind
	Type of control condition (e.g. sham stimulation; control group; control task)
	Online or offline delivery of neuromodulation (relative to cognitive task)
Measure of cognitive function	Cognitive task/paradigm
	Cognitive function(s) being measured
	Description of stimulus/cue type (if appropriate)
	Number of trials; task length
	Counterbalancing/order of conditions (if appropriate)
	Time of task relative to stimulation
Results	Descriptive statistics for condition/group means
	Test statistics for condition/group comparisons
	*P* values for condition/group comparisons
	Effect sizes for condition/group comparisons
	95% confidence intervals for condition/group comparisons

Using narrative review, we will evaluate the evidence that neuromodulation is effective in modulating cognitive functions in individuals who exhibit addictive behaviour. Evidence will be reviewed for different cognitive domains separately (e.g. risk-taking, impulsivity, attention). For each domain, we will review evidence for and against the effectiveness of neuromodulation techniques in modulating performance in that domain. In addition to final outcome (i.e. changes in performance associated with neuromodulation), we will assess and compare between studies the specific cognitive tasks that were used, the populations studied, the type and parameters of neuromodulation administered, and any other pertinent characteristics of the examined studies. The quality of studies and previously established risk of bias will be considered when weighing the evidence. After discussing the data pertaining to each cognitive area in turn, we will summarize our findings and identify areas for further research.

## Discussion

The proposed review will present the first systematic analysis of studies that have examined the effects of neuromodulation on cognitive functions in addictive populations. Neurocognitive deficits across a number of cognitive domains are a hallmark feature of SUAD, with deficits in impulse control such as response inhibition (e.g. [16]) and delay discounting [2, 23, 26] being particularly a characteristic of pathological substance use and gambling disorder. Collectively, these cognitive deficits contribute to numerous negative outcomes for individuals with SUAD, including loss of control, risky behaviours leading to injury or other negative consequences, and poor treatment outcomes. Therefore, the potential of using non-invasive neuromodulation techniques to alter neural processing and reduce impairments is an especially promising approach. Before techniques such as tDCS and TMS can be widely implemented as interventions for SUD, a critical appraisal of the extant literature is needed. Thus, this review fills an important need for both addictions’ researchers and clinicians.

## Abbreviations

PFC: Prefrontal cortex
PRISMA: Preferred Reporting Items for Systematic Reviews and Meta-Analyses
SUAD: Substance use and addictive disorders
tDCS: Transcranial direct current stimulation
TMS: Transcranial magnetic stimulation
